# Fatty Acid Signaling: The New Function of Intracellular Lipases

**DOI:** 10.3390/ijms16023831

**Published:** 2015-02-10

**Authors:** Zuzana Papackova, Monika Cahova

**Affiliations:** Centre for Experimental Medicine, Department of Metabolism and Diabetes, Institute for Clinical and Experimental Medicine, Prague 140 21, Czech Republic; E-Mail: zuzana.papackova@ikem.cz

**Keywords:** free fatty acids, intracellular signaling, hormone-sensitive lipase, adipose triacylglycerol lipase, lysosomal lipase

## Abstract

Until recently, intracellular triacylglycerols (TAG) stored in the form of cytoplasmic lipid droplets have been considered to be only passive “energy conserves”. Nevertheless, degradation of TAG gives rise to a pleiotropic spectrum of bioactive intermediates, which may function as potent co-factors of transcription factors or enzymes and contribute to the regulation of numerous cellular processes. From this point of view, the process of lipolysis not only provides energy-rich equivalents but also acquires a new regulatory function. In this review, we will concentrate on the role that fatty acids liberated from intracellular TAG stores play as signaling molecules. The first part provides an overview of the transcription factors, which are regulated by fatty acids derived from intracellular stores. The second part is devoted to the role of fatty acid signaling in different organs/tissues. The specific contribution of free fatty acids released by particular lipases, hormone-sensitive lipase, adipose triacylglycerol lipase and lysosomal lipase will also be discussed.

## 1. Introduction

Fatty acids are vital components of, essentially, all known organisms. They are important substrates for oxidation and production of cellular energy as well as precursor molecules for all lipid classes, including those that form biological membranes. On the other hand, free fatty acids (FFA) can become harmful to cells when present even at relatively low concentrations and thus they must be released under tight metabolic control. FFAs act as powerful signaling molecules, which regulate numerous cellular processes associated with lipid metabolism and which ensure precise matching of FFA release and demand. FFAs and their derivatives may regulate intracellular processes at several levels—gene transcription activation, post-transcriptional modification of proteins (*i.e.*, acylation) and direct modulation of enzyme activities as co-activators. In this review we will focus on the mechanisms of intracellular fatty acid signaling at the level of gene expression. We will further examine the role of fatty acid signaling in different organs.

## 2. Fatty Acids as Signaling Molecules: The Effect of Structure

Depending on the presence of double bonds, fatty acids are classified into three main groups: (1) saturated fatty acids (SFA), which do not contain double bonds; (2) monounsaturated fatty acids (MUFA); which contain one double bond and (3) polyunsaturated fatty acids (PUFA), which contain at least two double bonds. According to the position of the first double bond from the methyl end of the FFA molecule, PUFAs are described as *n-3*, *n-6* or *n-9*. After binding to their intracellular or membrane receptors, FFAs can initiate a wide variety of metabolic pathways, the particular effects of which depend greatly on the level of saturation. The quantity and quality of dietary lipids are known to have an impact on the composition of tissue lipids both in rodents [1] and humans [2,3]. Nevertheless, the composition of intracellular TAG stores also depends on the action of other enzymatic systems and reflects the activities of enzymes responsible for synthesizing, desaturating and elongating FFAs [4].

### 2.1. Free Fatty Acid Signaling Independent of Gene Transcription Modulation

The indirect effects of FFAs are made up of many diverse mechanisms. The fatty acyl composition of membrane lipids determines membrane fluidity, lipid raft structures and, as a consequence, signal transduction [5,6], where membrane fluidity increases according to the higher content of unsaturated fatty acids (SFA < MUFA < *n-6* PUFA < *n-3* PUFA). For example, SFA stimulates TLR2 and TLR4 signaling pathways and Src-family kinases, while *n-3* PUFA antagonizes this response [7] by both modifying micro-domain localization of signaling proteins and altering their functionality [8]. Membrane fatty acids, excised from phospholipids by cellular phospholipases, act as substrates for the synthesis of numerous pro- and anti-inflammatory eicosanoids, resolvins and protectins [9]. In general, *n-6* PUFAs are predominantly associated with the production of pro-inflammatory cytokines. By contrast, *n-3* PUFA-derived molecules exert an anti-inflammatory effect [10]. *n-3* PUFA may directly modulate the activity of the key pro-inflammatory transcription factor NF-κB by preventing NF-κB activation through the process of impeding I-κB phosphorylation [11]. SFAs, particularly palmitic acid and stearic acid, have been shown to induce endoplasmic reticulum (ER) stress and apoptosis; however, this has not been observed in case of oleic or linoleic acid [12]. SFAs have recently been proposed as triggers of the NLRP3 inflammasome, a molecular platform that mediates the processing of IL-1β in response to infection and stress conditions. In contrast, *n-3* PUFAs, but not *n-6* or *n-9*, inhibit NRLP3 inflammasome activation in various settings [13].

### 2.2. FFA Signaling Mediated by Nuclear Transcription Factors

Fatty acids may regulate cellular metabolism by acting as agonistic ligands of different nuclear receptors. When activated, these factors bind to the promoter regions of specific genes and selectively affect their transcription. In general, SFAs and PUFAs perform opposite actions; SFA are mostly associated with adverse effects on cell metabolism, while MUFAs and PUFAs have beneficial effects. SFAs are associated with metabolic pathways which promote steatosis. SFAs stimulate the expression of genes involved in the SREBP pathway (SREBP1, sterol-regulatory element binding protein and the cleavage-activating protein, site 1 protease) and cholesterol homeostasis (sterol *O*-acyltransferase) [14]. Recent evidence in mice has shown that SFAs may mediate their hyperlipidemic effects via peroxisome proliferator-activated receptor g coactivator-1b (PGC-1b) and the co-activation of SREBP. PGC-1b expression has also been shown to be greatly increased in cultured rat primary hepatocytes treated with individual SFAs, in particular after incubation with palmitic acid [15]. In contrast, *n-3* PUFAs function as feed-forward activators of fatty acid oxidation and feedback inhibitors, which prevent the production of new fatty acids including PUFA [16]. Challenging cells with a fatty acid that is typically at very low abundance in the tissue, such as eicosapentanoic acid (EPA), promotes a robust response, in particular, via nuclear transcription factors that target gene expression. In contrast, challenging cells with highly abundant fatty acids, *i.e.*, oleic acid, has little effect [7].

## 3. Fatty Acid-Activated Receptors

FFA-binding nuclear transcription factors belong to several families, including peroxisome proliferator-activated receptors (PPARs), sterol regulatory element-binding proteins (SREBPs), liver X receptor (LXR), farnesoid X receptor (FXR) and nuclear factor (erythroid-derived 2)-like 2 (Nrf2). Due to extremely low FFA solubility in the cytosol, translocation of FFAs from the plasma membrane to the site of utilization is mediated by cytoplasmic fatty acid binding proteins (FABPs) that serve as intracellular acceptors of FFAs. It has been proposed that FABPs sequester and/or distribute the ligands in order to regulate signaling processes and enzyme activities [17]. FFAs also specifically interact with several receptors localized in the plasma membrane (Toll-like receptors, the GPR superfamily), but these receptors respond to extracellular fatty acids that are not subjects of this review.

### 3.1. Peroxisome Proliferator-Activated Receptors (PPARs)

The most extensively studied fatty acid-dependent transcription factors are peroxisome proliferator-activated receptors (PPAR). PPARs belong to the family of nuclear hormone receptors that are activated by small lipophilic molecules and are characterized by unique tissue expression patterns [18,19]. Almost all fatty acids activate PPARs to a certain degree in transient assay systems, displaying general preferences for long chain PUFAs [20]. Concerning activation potency, *n-3* PUFA EPA and docosahexanoic acid (DHA) are more potent as *in vivo* co-activators than *n-6* fatty acids [21]. PPAR signaling belongs to the pathways critical for metabolic adaptation for starvation. The main mechanism of this adaptation is the use of long-chain fatty acids (LCFAs) as the main source of energy and minimizing glucose utilization [22].

PPARα and PPARδ are found predominantly in oxidative tissues such as brown adipose tissue, cardiac muscle, skeletal muscle and liver and probably share common target genes. They regulate genes involved in substrate delivery, substrate oxidation and oxidative phosphorylation. PPARα is a master regulator of hepatic lipid catabolism, as it induces the expression of numerous genes involved in mitochondrial and peroxisomal fatty acid oxidation. It plays a role also in the regulation of inflammation-related pathways and even in the modulation of glucose metabolism [23]. In one study, PPARα null mice grew apparently normally when fed *ad libitum*, but during food deprivation PPARα knockout mice exhibited lower blood glucose and were unable to induce sufficient fatty acid oxidation and ketogenesis [24]. PPARδ, although expressed ubiquitously, seems to play a critical role, especially in skeletal muscle metabolism [25]. Activation of PPARδ in the skeletal muscle is considered to be a primary mechanism, which increases the reliance of muscle cells on LCFAs and the attenuation of glucose utilization during fasting and prolonged exercise [26]. In addition to inducing genes for LCFA oxidation PPARδ may also play a role in mitochondrial biogenesis and muscle fiber remodeling [22]. Several reports indicate that the activation of PPARδ during fasting and endurance training is dependent on incoming (extracellular) FFAs [27].

PPARγ-1 and PPARγ-2 act as nutrient sensors, whose main roles are to finely tune metabolic homeostasis when faced with different nutritional states. In adipose tissue, they sense non-esterified LCFAs and induce pathways to store them as TAG. When stimulated, PPARγ up-regulates gene encoding proteins involved in fatty acid transportation (CD36, fatty acid-binding protein 4) [28,29], in the suppression of lipolysis (perilipin) [30] and in the shift in glucose metabolism from oxidation (pyruvate dehydrogenase inhibition) [31] to the provision of trioses necessary for fatty acid esterification (phosphoenolpyruvate carboxykinase and glucokinase up-regulation) [32,33]. Ablation or impairment in the function of PPARγ in adipose tissue (PPARγ-ATKO) produces an insulin-resistant lipodystrophic phenotype. Some murine models suggest that PPARγ is expressed outside adipose tissue and plays an important role in lipid homeostasis [34]. Liver-specific PPARγ knockout has been shown to reduce hepatic triacylglycerol storage both in extreme obesity (ob/ob mice) [35] as well as in lipodystrophy [36] at the expense of the worsened serum lipid profile and impaired insulin sensitivity. These data indicate that in specific situations of lipid excess, liver PPARγ activity may help to protect other tissues from lipid exposure. The generation of macrophage PPARγ-specific knockout mice revealed the importance of this transcription factor in the regulation of genes involved in cholesterol homeostasis in macrophages by ABC transporters [37]. PPARγ is also expressed in β-cells where it has a significant impact on islet morphology [38]. PPAR-γ activation could induce insulin secretion in β-cells [34].

### 3.2. Sterol-Regulatory Element Binding Protein 1 (SREBP1)

Sterol-regulatory element binding proteins belong to a family of basic helix-loop-helix leucine zipper transcription factors. SREBP1 preferentially activates genes involved in *de novo* lipogenesis, while SREBP2 tends to activate genes involved in cholesterol synthesis and uptake [39]. Nutrient control of SREBP nuclear abundance is mediated by transcriptional and post-transcriptional mechanisms. SREBPs are synthesized as precursors and must be cleaved prior to transport into the nucleus. In the nucleus, they bind to specific sequences (insulin response elements) in promoters of target genes [40]. Once bound, SREBs are phosphorylated and then interact with co-activators recruited to promoters; these events stimulate gene transcription [41]. Phosphorylation of SREBPs also acts as a signal for ubiquitination and proteasomal degradation [42]. PUFAs, but not SFAs or MUFAs, potently suppress genes involved in *de novo* lipogenesis [43], FFA desaturation [44] and elongation [16] in the liver by the attenuation of SREBP1c activity due to the suppression of proteolytic activation and decrease of mRNA stability [45]. Since desaturases and elongases are involved in PUFA synthesis, PUFAs become feedback inhibitors of their own as well as of MUFA synthesis (52 Jump). In addition, DHA (but no other PUFA), stimulates the removal of mature nuclear SREBP1 via a mechanism that depends on the 26S-proteasome and extracellular signal-regulated kinase [46]. The nuclear form of SREBP-1 is more sensitive to DHA regulation than the SREBP-1 precursor [7]. One hypothesis for explaining the mechanism of PUFA action on SREBP1c supposes the involvement of the liver X receptor α (LXR), but its role is still debatable [47]. The nuclear hormone receptors, retinoid X receptor and LXR, function as heterodimers in order to up-regulate SREBP-1c in response to cholesterol overloading. This process is possibly a way to increase the supply of unsaturated fatty acids needed for cholesterol storage and esterification [48]. PUFAs, as well as MUFAs and SFAs, inhibit the induction of SREBP-1c by the LXR agonist [49], which supports the hypothesis that LXR mediates fatty acid signaling as a step towards SREBP-1c. On the other hand, the suppressive effect of fatty acids on *de novo* lipogenesis is restricted to PUFA, whereas MUFAs and SFAs have no effect. Furthermore, PUFA inhibits lipogenesis by compromising SREBP-1c mRNA stability, whereas the SREBP-1c transcription rate is not affected [43,45]. With respect to these findings, a full explanation for the mechanism of PUFA’s effect on SREBP-1c requires further experiments.

### 3.3. Nuclear Factor-Erythroid 2-Related Factor 2 (NRF2)

Dietary PUFAs intake causes rapid up-regulation of numerous gene encoding proteins involved in oxidative stress response in several organs, which most likely indicates an adaptive mechanism aimed at preventing cellular lipotoxicity [50]. The important genes involved in the anti-oxidative response possess an antioxidant response element (ARE) sequence in their promoters that binds to NRF2. Regulation of NRF2 activity represents a critical step in the initiation of the cellular antioxidant response to reactive oxygen species [51]. The effect of dietary PUFAs, particularly DHA and EPA on the expression of genes involved in the oxidative stress response are most likely mediated by specific fatty acid oxidation products via NRF2 [52]. Oxidized *n-3* fatty acids stabilize NRF2 and increase the endogenous expression of NRF2 target genes.

### 3.4. Farnesoid X Receptor (FXR)

FXR is a nuclear receptor for bile acids and, in addition to bile acid synthesis regulation, it plays an important role in regulating lipid and carbohydrate metabolism and inflammatory responses. Activation of FXR lowers hepatic triacylglycerol content, inhibits inflammation and promotes liver regeneration [53,54]. Importantly, DHA, as well as arachidonic acid and linoleic acid, are ligands for FXR [55].

### 3.5. Fatty Acid Binding Proteins (FABPs)

Until now, at least nine members of FABP have been identified [56]. Different members of the FABP family exhibit unique patterns of tissue expression (liver (L)-, intestinal (I)-, heart (H)-, adipocyte (A)-, epidermal (E)-, ileal (Il)-, brain (B)-, myelin (M)- and testis (T)-FABPs) and are expressed most abundantly in tissues involved in active lipid metabolism. However, no FABP is exclusively specific for a given tissue or cell type, and most tissue express more than one FABP isoform [17]. The different FABPs display different degrees of ligand specificity. L-FABP and E-FABP do not discriminate between SFA, MUFA or PUFA, while H-FABP (expressed predominantly in the heart and skeletal muscles) is selective for *n-6* PUFAS, such as arachidonic acid [57]. B-FABP is highly selective for *n-3* PUFA, such as DHA [58]. FABPs appear to access the nucleus under certain conditions and potentially transport fatty acids to transcription factors. For example, L-FABP interacts with PPARα and PPARγ, but not with PPARδ or RXRα. In murine hepatocytes, L-FABP acts as a positive regulator of PPARα activity, with nuclear receptor activity being strictly dependent on intracellular L-FABP concentration [59]. A-FABP and E-FABP selectively enhance the activities of PPARγ and PPARβ, respectively, and these FABPs massively relocate to the nucleus in response to selective ligands of the PPAR isotype, which they then activate [60]. L-FABP stimulated by unsaturated fatty acids, especially linoleic acid, has been reported to promote hepatocyte proliferation [61]. In the central nervous system, FABPs show differential expression according to the developmental stage. E- and B-FABPs actively take part in embryonic neurogenesis, while H-FABP appears to be engaged in the maintenance of normal neuronal functions [62]. L-, H-, A- and E-FABP are controlled by PPARs transcription factors [17]. Taken together, these findings indicate that FABPs function as a mandatory cytosolic gateway for transportation of the activators into the nucleus and enable PPARs’ activation by their specific ligands.

## 4. The Origin of Signaling FFA

Intracellular FFA may originate either from hydrolysis of cytosolic TAG stores or may be imported from the plasma FFA pool. Alternatively, they may be generated by lipoprotein lipase activity from circulating lipoproteins and transported into the cell or synthesized *de novo*. All of these distinct pools may serve as a source for signaling molecules, but according to the present state of knowledge they differ with respect to tissue and metabolic stimuli. What still remains unresolved is the question of whether these fatty acids directly serve as nuclear receptor ligands, or if they need to undergo an esterification/hydrolysis cycle first. If the latter is true, the lipid droplets (LD) not only become passive energy-storage particles but also reservoirs of specific lipid mediators. FFAs are released from stored TAG via lipolysis and some evidence indicates that the release of individual fatty acids from adipose tissue is selective, at least in the case of adipose tissue. Raclot and Groscolas [63] reported that, compared to the source TAG, released FFAs were enriched in some PUFAs and depleted in long-chain SFAs and MUFAs. For a given number of double bonds, mobilization decreases with increasing chain length, whereas it escalates with increasing unsaturation for a specific chain length. Thus, FFAs are not mobilized in direct proportion to their content in TAG but selectively according to molecular structure [64].

The enzymes responsible for intracellular TAG hydrolysis, lipases, not only ensure energy substrate release, but actively regulate energy metabolism of the cell. So far, the following lipases have been described: hormone sensitive lipase (HSL), adipose triglyceride lipase (ATGL), monoglyceride lipase (MGL), lysosomal lipase (LAL) and carboxyl esterase 3/triglyceride hydrolase-1 (Ces3/TGH). The challenging and still unresolved issue remains the question of whether FFA, derived by individual lipases, has distinct signaling functions. By employing a DNA microarray method, Pinent *et al.* [65] identified biological processes that are modulated in different tissues—white adipose tissue (WAT), brown adipose tissue (BAT), the skeletal muscle, cardiac muscle and liver—of HSL-knockout (HSL-KO) and ATGL-KO mice. Although genetic ablation of ATGL and HSL altered the transcript levels of a large number of genes, only one biological process was modulated in the same way in both mouse models, namely the down-regulation of fatty acid metabolism in BAT. The most pronounced modulation of biological processes was observed in ATGL-KO in the cardiac muscle, in which a concerted down-regulation of transcripts associated with oxidative pathways was observed. By contrast, in HSL-KO mice the most marked changes were seen in the skeletal muscle, namely, alterations in transcript levels, which reflect a change of energy source from lipids to carbohydrates. These findings suggest that ATGL and HSL differentially modulate biological processes in individual metabolic tissues. On the other hand, it may help to identify the lipase that acts as the prevailing lipolytic enzyme in any particular tissue. A more detailed insight into this problem has been provided by studies using models with ATGL, HSL or LAL deficiency.

## 5. The Different Roles of Individual Lipases

Deleting genes that encode ATGL, HSL, LAL or Ces3/TGH in mice results in striking phenotypic differences, which suggests that these lipases have distinct roles [65]. HSL-deficient mice are non-obese [66,67] and are resistant to diet-induced obesity [68]. The adipose tissue phenotype of these mice includes reduced abdominal fat mass and marked heterogeneity in adipocyte size in both white (WAT) and brown (BAT) adipose tissue. The lipolytic rate in isolated HSL knockout (HSL-KO) adipocytes is impaired after β-adrenergic stimulation, but it remains normal under basal conditions [69]. HSL-deficient mice are glucose-intolerant and secrete less insulin than normal mice in response to a rise in blood glucose [70]. Unlike HSL-KO mice, ATGL knockout (ATGL-KO) mice show increased TAG deposition in several tissues and exhibit mild obesity caused by enlarged adipose lipid droplets. Notably, severe fat accumulation in the cardiac muscle in these mice leads to cardiac insufficiency and premature death. Other notable features of ATGL-deficient mice are increased glucose tolerance, increased insulin sensitivity, and an increased respiratory quotient during fasting, which suggests that glucose is used as a metabolic fuel [44]. LAL knockout (LAL-KO) mice appear normal at birth and survive until adulthood but their lifespan is shortened (7–8 months). Massive storage of TAG and cholesterol esters is observed in the adult liver, adrenal glands and small intestine. At 6–8 months of age, the LAL-KO mice have completely absent inguinal, gonadal and retroperitoneal white adipose tissue [71]. LAL is localized exclusively within lysosomes and it has been traditionally associated only with the degradation of lipoprotein particles internalized via endocytosis. Intracellular lipids have not previously been considered to be an LAL substrate, but the similarities between lipolysis and autophagy, a process that ensures transportation of intracellular particles into lysosomes, together with the existence of lysosomal lipase, suggest a possible link between these two pathways. Both lipolysis and autophagy are essential catabolic pathways, which become activated in response to nutrient deprivation. They fall under identical hormonal control and are either inhibited by insulin or activated by glucagon [72]. An inter-relationship between the two processes has been demonstrated by the finding that autophagy mobilizes lipids from lipid droplets for metabolic purposes through a process termed “lipoautophagy” [73]. Ces3/TGH is localized in the endoplasmic reticulum lumen and it has been suggested that it plays a role in in the provision of substrates for the assembly of apoB-containing lipoproteins [74]. In one study, global and liver-specific inactivation of Ces3/TGH decreased plasma, very-low density lipoprotein (VLDL), TAG and VLDL cholesterol concentrations, without significantly elevating hepatic steatosis even when mice were challenged with a Western type diet. Conversely, another study found that Ces3/TGH-deficient mice exhibited increased plasma ketone bodies and hepatic fatty oxidation [75,76]. Furthermore, this enzyme seems to be involved in regulating the rate of lipid transfer into preformed cytosolic lipid droplets [77]. Taken together, these data put forward an interesting interpretation, which is that different lipid pools may result in different metabolic uses and that they are accessible by different lipases. The regulatory mechanisms of this hypothetical phenomenon are not known.

## 6. Lipase Activities Are Modulated via Interaction with Regulatory Proteins

The physiological activity of the three main intracellular lipases (HSL, ATGL, LAL) is regulated by interaction with several other proteins, which ensures the fine adjustment of the lipolytic rate to the actual metabolic needs of the organism. Furthermore, all lipolytic enzymes are confronted with the principal problem of how to move into close proximity of both the hydrophobic substrate and the enzyme. Full enzymatic function under these conditions is often dependent on, and regulated by, co-factor proteins.

### 6.1. Hormone-Sensitive Lipase

A key feature of HSL is its regulation via reversible phosphorylation by cAMP-dependent protein kinase A (PKA) in response to β-adrenergic stimulation [78]. Interestingly, *in vitro* phosphorylation of purified HSL with PKA results only in a modest (2-fold) increase in the intrinsic catalytic activity of the hormone, whereas isolated adipocytes, when stimulated with β-adrenergic receptor agonists, exhibit a 30–50-fold increase in lipolysis [79]. This discrepancy indicates that there is an additional mechanism that ensures the gross lipolytic response to hormonal stimuli. Lipid droplets in fully differentiated adipocytes are covered with perilipins A and B [80]. Under basal conditions, perilipins limit the ability of HSL to associate with the lipid droplet surface. Recent studies have revealed that, upon adrenergic stimulation, both HSL [81] and perilipin [82] are phosphorylated concomitantly and HSL is translocated to the lipid surface, where it replaces phosphorylated perilipin. Besides PKA, perilipins could be phosphorylated by other kinases including ERK kinase [83], cGMP-dependent kinase [84] and cAMP-activated protein kinase [85], which could enable the integration of numerous metabolic signals. Genetic ablation of perilipin induces alterations in the gene expression pattern, up-regulates oxidative pathway-related genes and down-regulates genes involved in lipid biosynthesis [86].

### 6.2. Adipose Triglyceride Lipase

Recent reports have revealed that ATGL activity is, similar to HSL, activated by hormonal signals [87]; however, the underlying molecular mechanisms are different. First, ATGL occurrence on LD is similar both in basal and stimulated states [88]. Second, ATGL is not a PKA target [89]. And finally, ATGL is grossly activated by comparative gene identification-58 (CGI-58), whereas it has no effect on HSL activity. CGI-58 binds to LD by interaction with perilipin A in a hormone-dependent way [90]. In an unstimulated state, CGI-58 binds to perilipin A, but it is unable to activate ATGL. Following hormonal stimulation, perilipin is phosphorylated at several serine residues, whereas CGI-58 dissociates from perilipin, interacts with ATGL and activates TAG hydrolysis [89]. Several proteins have been shown to inhibit ATGL enzymatic activity. Adipocyte differentiation related protein (ADRP) is abundant on the surface of small lipid droplets, a characteristic of preadipocytes, [91] and it is also expressed on the LD surface in non-adipose tissue that lacks perilipin, such as the muscle or liver [80]. The role of ADRP in the regulation of ATGL activity has not been fully elucidated yet, but the negative effect of ADRP on ATGL access and TAG lipolysis has been demonstrated in HEK293 cells and other cell lines [92]. ATGL activity in adipose tissue can also be selectively inhibited by the G0S2 protein (a product of the G0/G1 switch gene 2) [93]. Studies employing co-expression approaches have shown that G0S2 is able to prevent turnover of LDs mediated by ATGL. More importantly, it has been determined that G0S2 directly interacts with ATGL and is capable of inhibiting the TG hydrolase activity of ATGL, even under CGI-58 co-activated conditions [94].

The pooled data suggest that LDs are embedded by a complex network (lipolysome), which consists of lipases and numerous modulators of lipolytic activity [90]. Storey *et al.* [95] have proposed an interesting mechanism that explains the functional interaction of LD-coating proteins. Their hypothesis is based on the biophysical characteristics of the phospholipid monolayer on the LD surface. Adipocyte LD proteins involved in lipolysis, including perilipins, HSL and AGTL, target areas in the phospholipid monolayer that are highly organized and rigid, similar in structure to localized areas of the plasma membrane where cholesterol and fatty acid uptake and efflux occur. In contrast, the ADRP-enriched LD proteins contain a more fluid monolayer membrane, reflecting decreased polarization and lipid ordering.

### 6.3. Lysosomal lipase

Lysosomal lipolysis of cytosolic TAG droplets mediated by LAL depends on the successful delivery of autophagolysosomes involved in the process called auto(lipo)phagy [73]. During this process, a portion of cytosol is engulfed by a double-membrane structure, called an autophagosome, which fuses with a lysosome, whose enzymes degrade the cellular constituents sequestered in the autophagosome [96]. The regulation of this process is complex as it is controlled by the coordinated actions of autophagy-regulated genes (Atgs), over 30 of which have been identified in yeast and humans [97]. The determinants of autophagic initiation on the surface of LD remain unknown. Poly-ubiquitination has been detected in polarized areas of LD, in part resulting from the accumulation of clusters of poly-ubiquitinated apolipoprotein B (ApoB) on their surfaces [98]. It seems that ApoB can undergo both proteasomal and lysosomal degradation, as proteasome inhibition causes an increase of autophagic vacuole abundance and ApoB content in lysosomes. Whether or not the lysosomal degradation of ApoB occurs as a result of the activation of lipophagy, or how the accumulation of this protein contributes to the initiation of the process, requires future investigation [99].

## 7. Thioesterases Regulate the Intracellular Unconjugated FFA *vs.* Fatty Acyl-CoA Ratio

It is important to note that unconjugated FFAs can function as signaling molecules. Nevertheless, FFAs released by intracellular lipases are readily converted by long-chain acyl-CoA synthetases into fatty acyl-CoA (FA-CoA) and enter various metabolic pathways, *i.e.*, β-oxidation or TAG synthesis. The reverse reaction, *i.e.*, hydrolysis of FA-CoA, is catalyzed by members of the acyl-CoA thioesterase (Acot) gene family [100]. The physiological functions of these enzymes are far from being completely understood. The proposed roles for these enzymes include (1) control of the intracellular balance between fatty acyl-CoAs and free fatty acids, *i.e.*, the availability of FFA moieties either for metabolic utilization or signal transduction; (2) the intracellular and intra-organelle concentrations of Coenzyme A; and (3) the availability of fatty acids for biosynthesis of inflammatory mediators [101,102]. Several Acot family members have been categorized as a thioesterase superfamily (*Them*) [100]. Them-1 is highly expressed in BAT and is regulated by both ambient temperature and food consumption. Recently published research indicates that Them-1 functions in order to decrease energy consumption in BAT, leading to the conservation of calories during exposure to cold. Furthermore, Them-1 seems to promote obesity-related inflammation and endoplasmic reticulum (ER) stress in WAT via the generation of pro-inflammatory lipid mediators/FFA [103]. Them-2 is enriched in oxidative tissues such as the liver, heart, kidney and BAT. It is involved in the regulation of fatty acid and glucose metabolism in the liver, particularly by limiting β-oxidation via PPARα and HNF4α signaling pathways [104]. In BAT, Them-2, possibly in cooperation with Them-1, controls the dynamic inter-conversion of long chain FA-CoAs and FFA in order to regulate adaptive thermogenesis and to suppress adaptive increases in energy expenditure [104]. Them4 is expressed in the skeletal muscle, testis, uterus, brain and kidney and attenuates insulin and IGF-1 signaling via the reduction of Akt activity [105]. It also contributes to the regulation of mitochondrial dynamics, particularly the fission process [106]. This enzyme has been shown to hydrolyze a wide spectrum of substrates including medium- to long-chain fatty acyl CoAs [107], but it is still unclear whether a relationship exists between the enzymatic activity of Them4 and its regulatory activity in cell signaling [100]. Them-5 is a mitochondrial protein localized to the matrix [108] and exhibits enzymatic selectivity for medium- to long-chain FA-CoA. Them-5 plays an important role in the remodeling of mitochondria. The loss of Them-5 in *Them-5*^−*/*−^ mice has been associated with decreased fatty acid oxidation and TAG accumulation in the cytosol. All these data indicate that Them proteins play a key role in the regulation of numerous metabolic processes via intracellular fatty acid channeling.

## 8. Fatty Acid Signaling in Different Tissues

### 8.1. Liver

The liver is capable of eliminating large amounts of FFAs from circulation by storing them in the form of TAG. Furthermore, it is the only organ capable of transforming FFAs into ketone bodies, which provide an energy source for the brain in periods of starvation. The central role of the liver in lipid and energy metabolism makes the well-balanced regulation of these processes vitally important. In the liver, the metabolic response is strongly dependent on the saturation of the signaling FFA molecule. The liver appears to use its highly unsaturated fatty acid status as a nutrient sensor to determine whether fatty acids are to be stored or oxidized. A diet rich in *n-3* and *n-6* PUFA (*i.e.*, 2%–5% of energy is provided in the form of PUFA) leads to the suppression of lipogenic genes. This is achieved by the inhibition of SREBP-1c transcription activity and the induction of genes involved in FFAs oxidation via stimulation of PPARα [109]. Conflicting results concerning the abilities of different PUFA to induce FFAs partitioning have been reported. According to Clarke [40], only dietary *n-3* and *n-6* PUFAs and their respective products from the ∆6-desaturase pathway are able to modulate hepatic lipid metabolism, whereas elongated and desaturated products of SFA and MUFA have no effect. In contrast, Chakravarthy *et al.* [110] demonstrated that products of fatty acid synthase, *i.e.*, C16:0 and its derivatives, can effectively modulate at least some of the PPARα target genes.

Another line of evidence stresses the importance of FFA origin with regard to their signaling properties. Using an ATGL gain- and loss-of-function mice model, Ong *et al.* [111] demonstrated that ATGL-derived FFA affected β-oxidation, but had no effect on very low-density lipoprotein secretion. In mice with suppressed ATGL expression, the effect on fatty acid oxidation coincided with decreased expression of PPARα and its target genes. The FFA composition of intracellular TAG was not determined in these studies. Another point of view indicates that both FFAs released by lipolysis and FFAs synthesized *de novo* modulated gene expression, but these distinct pools do not seem to be fully interchangeable in the liver. Chakravarthy *et al.* [110] reported that mice with liver specific knockout of fatty acid synthase (FASKOL) and who were administered a fat-free diet, exhibited a similar phenotype to PPARα-deficient mice, but only some of the affected metabolic processes could be offset by dietary fat. When lipids were provided in the diet, FASKOL mice were protected from the hypoglycemia/steatohepatitis phenotype, which suggests that dietary fatty acids are able to activate a pool of PPARα and promote gluconeogenesis as well as fatty-acid oxidation. Regardless of diet, FASKOL mice have low hepatic stores of cholesterol that are normalized by pharmacological PPARα activation. This suggests that “new” fatty acids produced by fatty acid synthase have access to a separate pool of PPARα and are capable of stimulating cholesterol biosynthesis in addition to gluconeogenesis and FFA oxidation.

Recently, Jha *et al.* [112] provided evidence that reveals the novel protective role of ATGL-derived FFA in combatting hepatic inflammation. The lack of ATGL increased susceptibility to a methionine-choline-deficient (MCD) diet and lipopolysaccharide (LPS)-induced inflammation in mouse liver, which was attributed to impaired PPARα DNA-binding activity. Notably, mice lacking HSL did not exhibit an aggravated response to MCD and LPS, which supports the hypothesis on specialized functions of HSL- and ATGL-released FFA in intracellular signaling.

### 8.2. White and Brown Adipose Tissue

Lipolysis and endogenous FFAs production belongs to the key metabolic processes occurring in adipose tissue. The released FFAs are exported out of the cell as energy substrates (in WAT) or used as a fuel for thermogenesis (in BAT). Numerous reports indicate that intracellular FFAs also act as an indispensable signal, which regulates the complex gene expression pattern that determines the WAT or BAT phenotype. In WAT, FFAs stimulate genes associated with TAG synthesis and adipogenesis in compliance with the main physiological function of this tissue. Amri [113] showed that FFAs stimulate the expression of the adipocyte-specific protein aP2. In contrast to the rapid effects of FFAs on gene expression in the liver, this process is much slower. Moreover, saturated and unsaturated FFAs are equipotent [114]. The effect of FFAs on aP2 expression requires proteosynthesis, which negates the direct action of FFAs on the aP2 gene, but supports the possibility that FFAs alter the synthesis of some other effector proteins [115]. PUFAs, especially DHA, are ligands for PPARγ2, an adipocyte-specific nuclear hormone receptor. Following the activation by fatty acids, induction of PPARγ2 increases the expression of the CCAAT/enhancer-binding protein α, leading to the activation of adipogenic genes [29]. The tissue-specific effect of FFA signaling may be demonstrated on the PEPCK gene, which is activated by PUFAs, particularly by DHA, in adipose tissue, but not in the liver [116]. On the other hand, PUFAs has been repeatedly shown to prevent excessive adipose tissue growth. Similar to the liver, PUFAs stimulate the expression of genes involved in FFA oxidation [117] and attenuate expression of some lipogenic genes, e.g., stearoyl-CoA desaturase 1 [118].

Furthermore, recent reports suggest that the main adipose lipases (HSL, ATGL) regulate distinct metabolic pathways in adipose tissue. The data derived from adipose-specific ATGL-KO mice suggest that ATGL-catalyzed lipolysis plays an important role in the plasticity of adipocytes, conceivably by providing ligands for binding/activation of PPARα. Adipose-specific ATGL-KO mice have been shown to exhibit signs of BAT to WAT-like tissue conversion. These mice also exhibited severely impaired thermogenesis with increased expression of WAT-enriched genes. However, they did display decreased BAT genes, including uncoupling protein 1 (UCP1), as well as reduced PPARα binding to their promoters [119]. No defects were observed in BAT morphology during embryonic development. Mottillo *et al.* [120] demonstrated that β-adrenoreceptor agonist-stimulated expression of thermogenic genes (peroxisome proliferator-activated receptor γ coactivator 1-α, PGC-1α; pyruvate dehydrogenase kinase 4, PPARα, UCP1) was down-regulated by pharmacological or genetic inhibition of both HSL and ATGL in isolated brown adipocytes. Conversely, treatments that increased endogenous fatty acid production elevated expression of oxidative genes. They further showed that ligands for PPAR-α and -δ, but not PPAR-γ, were generated on the lipid droplets’ surface after β-adrenergic stimulation and could transcriptionally activate PPAR-α and -δ.

Functional HSL seems to be essential for normal WAT development in mice, but not for BAT. Weight and TAG content were reduced in WAT but increased in BAT of HSL-KO mice. The expression of transcription factors associated with adipogenesis (PPARγ, CCAAT-enhancer-binding protein, C/EBPα), lipogenesis (SREBP-1c, diacylglycerolacyl transferase 1 and 2, fatty acid synthase, ATP citrate lyase), adipose differentiation markers (adipose differentiation-related protein, perilipin, lipoprotein lipase) and insulin signaling was suppressed in WAT of HSL-KO mice [68], but not in BAT. The authors concluded that the function of HSL in adipocyte differentiation may lie in supplying intrinsic ligands, FFAs, which activate the transcription factors, PPARγ and C/EBPα. The abnormalities that result from producing fatty acids prevent complete differentiation of WAT, resulting in alterations of adipocyte-derived hormones and cytokines with systemic manifestations. Interestingly, HSL deficiency does not represent an absolute obstacle for WAT differentiation, since WAT, although reduced, is present in all normal depots without any obvious evidence of lipodystrophy.

With respect to one of the most important catabolic process in WAT—hormone-stimulated lipolysis—FFAs seem to play an opposite regulatory role after acute and chronic β-adrenoreceptor stimulation. Stimulation with β agonists results in rapid elevation of protein kinase A (PKA) target genes (PGC1α, UCP1, neuron-derived orphan receptor 1). However, rather surprisingly, the mobilized FFAs negatively feeds back to PKA activity and limits the expression of PKA-target genes. Inhibiting HSL by pharmacological or genetic means potentiates the β-adrenoreceptor stimulation of PKA and its target genes expression [121]. In contrast, during chronic β-adrenergic stimulation, HSL-derived FFAs promote mitochondrial biogenesis, expand oxidative capacity and increase lipid droplet fragmentation [122]. These different responses to acute and chronic β receptor stimulation in WAT may be explained as adaptations, as a way to ensure that FFA production closely matches the prevailing metabolic demands of the organism and, concurrently, that the potentially harmful effects of FFA are minimized. In the case of an acute burst of lipolysis provoked by an actual need of energy, the modulatory effect of FFAs lead to the limitation of intracellular FFA utilization in favor of their extracellular export. During chronic β receptor stimulation, WAT is remodeled in order to re-direct the excessive FFA flow into oxidation/wasting, whereby FFA-detrimental effects are prevented.

### 8.3. Cardiac Muscle

Using a whole body ATGL-KO mice model, Haemmerle *et al.* [123] demonstrated that the activation of cardiac gene transcription by PPARα depends on lipolytic degradation of intracellular lipid stores and that this process requires ATGL hydrolytic activity. ATGL-KO mice exhibited decreased mRNA levels of PPAR-α and -δ target genes, which were associated with decreased Pgc-1α and Pgc-1β expression and defective mitochondrial oxidation. All metabolic derangements could be corrected either by cardiac-specific expression of the *Atgl* transgene or by pharmacological treatment with PPARα agonists. The normalization of cardiac steatosis in response to the PPARα agonist occurred without an increase of cellular lipolysis, which excludes the involvement of any other lipase. Interestingly, this beneficial effect in the cardiac muscle was not obtained by administering PPARδ agonists, even though this transcription factor is generally associated with increased fatty acid oxidation in the muscle and liver. Interesting data were derived from a model of inducible cardiomyocyte-specific ATGL deficiency (ic*Atgl*KO). In this model, ATGL was disabled in adult mice. Several weeks after *Atgl* ablation, ic*Atgl*KO mice exhibited markedly increased myocardial TAG accumulation, fibrotic remodeling and hypertrophy. Fatty acid oxidation capacity was diminished, but it was not paralleled by markedly impaired PPARα target gene expression, as the mild reduction of PPARα signaling was detectable only after prolonged (12 h) fasting [124]. In these mice, HSL activity was up-regulated, which could partially offset the effect of ATGL ablation. This hypothesis is in accordance with previously reported findings that cardiac-specific HSL overexpression protects against fasting- and diabetes-induced cardiac steatosis [125,126]. Nevertheless, normal HSL activity was not able to fully compensate for impaired fatty acid signaling in whole body ATGL-KO mice.

### 8.4. Macrophages

Macrophages are versatile cells that fulfill various functions in response to environmental changes. They are able to uptake significant amounts of FFAs from the environment and store them in the form of TAG and cholesterol esters. Macrophages express considerable amounts of active ATGL [127], even though the physiological relevance of HSL in these cells is still a matter of debate [78]. ATGL-KO macrophages adopt specific phenotypes, characterized by TAG accumulation even in the absence of exogenous lipid loading, which results in a cell morphology that resembles macrophage foam cells. Surprisingly, markedly decreased atherosclerosis has been observed in low-density lipoprotein receptor-deficient mice after transplantation with ATGL-KO bone marrow [128]. The observed attenuated lesion formation was associated with drastically reduced plasma levels of monocyte chemotactic protein-1 (an important chemoattractant for mononuclear cells), reduced migration rates of ATGL-KO macrophages and markedly diminished production of pro-inflammatory chemokine IL-6, due to impaired pro-inflammatory signaling. Aflaki *et al.* [129] showed that the migratory capacity of ATGL-KO macrophages was markedly reduced *in vivo* and *in vitro* as a result of reduced expression of adhesion molecules and defective actin dynamics. In conclusion, defective lipolysis in macrophages that lack ATGL favors an anti-inflammatory M2-like macrophage phenotype, which implies that endogenously-released FFA plays a role in inflammatory signaling in macrophages.

FFA signaling in macrophages is dependent on the type of FFA. Recently, Varela *et al.* [130] reported that the nuclear receptor transcription factors PPARα, PPARβ/δ, and PPARγ as well as the PPAR-RXR transcriptional complex are all involved in sensing the proportion of MUFA oleic acid and SFA palmitic acid. Monounsaturated oleic acid binds to all three PPAR subtypes (PPARα > PPARβ/δ = PPARγ), whereas saturated palmitic acid binds with less efficiency to PPARα and PPARβ/δ (PPARα > PPARβ/δ) and not at all to PPARγ [131]. The increase in the MUFA oleic acid-to-SFA palmitic acid ratio in postprandial triacylglycerol-rich proteins was linearly correlated with a down-regulation of ApoB48 receptor mRNA and with a decrease of lipid accumulation.

### 8.5. Vascular Smooth Muscle Cells and Endothelial Cells

In contrast to macrophages, ATGL deficiency in endothelial and vascular smooth muscle cells (SMC) promotes a pro-inflammatory and pro-atherogenic phenotype. SMCs that do not express ATGL resemble neither the contractile (quiescent) state nor a proliferative (synthetic) phenotype of normal SMC, but they share some characteristics with senescent cells. ATGL-KO SMC exhibits a unique pattern of gene expression, particularly, down-regulated expression of vascular endothelial growth factor, type I collagen and transforming growth factor-β (TGF-β), while glucokinase, lipoprotein lipase and pro-inflammatory cytokine IL-6 are up-regulated. The distinct phenotype of ATGL-KO and its deviated gene expressions are corrected by an ectopic transfer of the wild-type *Atgl* gene. The regulatory mechanism of the ATGL-KO SMC phenotype is still unclear [132]. *In vitro* ATGL knockdown in the HAEC endothelial cell line, stimulated with TNFα, up-regulates the expression of the adhesion molecule, intracellular adhesion molecule (ICAM-1), resulting in enhanced monocyte adhesion [133]. These findings suggest that ATGL acts as an endogenous regulator of endothelial function and monocyte adherence.

### 8.6. Hypothalamus

Recent research has offered some novel and interesting insights into the role of FFA signaling in the regulation of appetite, energy balance and metabolism in the central nervous system. The important components of this neural network are located in the hypothalamic arcuate nucleus. It consists of neurochemically discrete and functionally antagonistic cells, including agouti-related peptide (AgRP) and pro-opiomelanocortin (POMC) neurons, which form a focal point for the integration of nutritional and metabolic cues, central and peripheral neural afferents and the action of adiposity hormones, such as leptin and insulin. The AgRP neurons promote food intake, in part through the release of AgRP, a physiological antagonist of melanocortin receptors. POMC neurons reduce food intake and increase energy expenditure by releasing the α-melanocyte-stimulating hormone.

Recent evidence indicates that one specific form of lipolysis—lipoautophagy—plays an important role in hypothalamic control of food intake and energy expenditure. Prior studies have shown that the brain, unlike other organs, is relatively resistant to the activation of autophagy in response to starvation. Nevertheless, in the hypothalamus, physiological periods of fasting and re-feeding regulate autophagy [134]. The fact that AgRP and POMC neurons profoundly affect feeding behavior and that autophagy seems to be essential for their proper function indicate that autophagy in the hypothalamus is regulated in a nutrient-dependent manner. This process is carried out via the mammalian target of rapamycin, AMP-activated protein kinase and class I phosphatidylinositol 3-kinase. Metabolites, such as free fatty acids, have also been implicated in the control of food intake as well as in the activation of autophagy in non-neuronal tissues such as hepatocytes [73]. The study by Kaushik *et al.* [134] proposes a unique mechanism for explaining how starvation increases AgRP expression via autophagy-derived neuronal FFAs. The authors observed that during fasting, hypothalamic fatty acid uptake and triacylglycerol synthesis *in vivo* is elevated and autophagy is stimulated. By inhibiting lysosomal lipolysis, it reduces fasting- and fatty acid-induced increases in orexigenic AgRP levels in hypothalamic cells and in primary neurons cultured under basal conditions [134]. These data suggest that the crucial role of autophagy in AgRP neurons is to provide signals (FFAs or their derivatives) that further stimulate AgRP expression. In this model, a crucial question arises—if intracellular fatty acids (or their metabolites) regulate AgRP gene expression, why is there a need for autophagy to generate neuron-intrinsic free fatty acids when starvation per se increases hypothalamic uptake of circulating free fatty acids? Kaushik *et al.* [134] shows that the immediate fate of starvation-induced fatty acid uptake is rapid triglyceride synthesis, possibly as a way to counteract cellular toxicity arising from a rapid surge of free fatty acids into the cell. The application of this two-step model (esterification/ lipolysis) could help to maintain a controlled availability of an endogenous pool of free fatty acids, functionally distinct from circulating free fatty acids during starvation. It may also shed light on the previous finding that neuronal availability of endogenous free fatty acids plays an important role in the regulation of feeding.

## 9. Conclusions

The findings presented in this review, as well as many others not noted due to space limitations, persuasively demonstrate the important role of intracellular fatty acids and/or their derivatives in the regulation of the pleiotropic spectrum of metabolic pathways. FFA may modulate such heterogeneous processes such as macronutrient metabolism, tissue differentiation, metabolic flexibility and inflammation. The great diversity of the targets indicates that there is cooperation with other “co-regulators”, which ensures the specificity of their actions. Although great advances have been achieved over recent decades in this field, there are still many unanswered questions regarding the exact details of FFA signaling. Uncertainty remains, for example, regarding the following issues: (1) Do FFAs need to undergo an esterification/lipolysis cycle prior to serving as an intracellular signal? Conversely, could “exogenous” FFAs, *i.e.*, those FFAs that are transported from extracellular spaces, directly affect intracellular signaling? (2) Is the function of intracellular FFAs as a signaling molecules source- or location-dependent? Do distinct TAG pools with specialized regulatory potential exist within cells? (3) Is it possible to influence FFA signaling via modulation of a particular lipase activity? Therapeutic strategies targeted at FFA signaling may lead in a promising direction in the treatment of metabolic disorders. Nevertheless, due to the extreme diversity of the metabolic processes affected by FFA signaling, an exact understanding of the underlying mechanisms is imperative and further research is needed.
